# Risk factors for incident delirium among urological patients: a systematic review and meta-analysis with GRADE summary of findings

**DOI:** 10.1186/s12894-020-00743-x

**Published:** 2020-10-27

**Authors:** L. Sanyaolu, A. F. M. Scholz, I. Mayo, J. Coode-Bate, C. Oldroyd, B. Carter, T. Quinn, J. Hewitt

**Affiliations:** 1 Division of Population Medicine, Cardiff University, Cardiff, UK; 2 Addenbrooke’s Hospital, Cambridge, UK; 3 Institute of Cardiovascular and Medical Sciences, University of Glasgow, Glasgow, UK; 4 Department of Biostatistics and Health Informatics, Institute of Psychiatry, Psychology and Neuroscience, King’s College London, London, UK; 5 Cochrane Skin Group, School of Medicine, The University of Nottingham, Nottingham, UK

**Keywords:** Delirium, Elderly, Urology, Epidemiology

## Abstract

**Background:**

Post-operative delirium is an important, yet under-researched complication of surgery. Patients undergoing urological surgery may be at especially high risk of POD, as they are often older, and interventions can be associated with conditions that trigger delirium. The main aim of this systematic review was to evaluate the available evidence for risk factors in this patient group.

**Methods:**

Five databases were searched (MEDLINE, Web of Science, EMBASE, CINAHL and PsychInfo) between January 1987 and June 2019. The Newcastle–Ottawa Scale was used to assess for risk of bias. Pooled odds ratio or mean difference (MD) for individual risk factors were estimated using the Mantel–Haenzel and inverse variance methods.

**Results:**

Seven articles met the inclusion criteria, giving a total population of 1937. The incidence of POD ranged from 5 to 29%. Three studies were deemed low risk of bias and four at a high risk of bias. Nine risk factors were suitable for meta-analysis, with age (MD 4.314 95% CI 1.597, 7.032 p = 0.002) and the clock drawing test (MD − 2.443 95% CI − 3.029, − 1.857 p < 0.001) having a statistically significant association with POD in pooled analyses.

**Conclusion:**

Delirium is common in urological patients. This review has identified a lack of studies in this surgical population, with wide heterogeneity and high risk of bias. It also highlights a number of potential risk factors for post-operative delirium, of which some are modifiable. However, the strength of evidence is weak at present and so future research should focus on assessing comparable risk factors in this patient group in order to inform future clinical practice.

**Review registration** The review protocol was prospectively registered with the PROSPERO database (reference CRD42017054613)

## Background

Delirium, derived from the Latin *deliriare* “go off the furrow”, describes a disturbance, or clouding, of consciousness and is diagnosed by fulfilling diagnostic criteria such as those proposed in the Diagnostic and Statistical Manual of Mental Disorders (DSM) [1, 2]. Additional features include agitation, hallucinations and disturbance in the sleep–wake cycle. Delirium is a multifactorial syndrome, associated with significant morbidity and mortality. A previous meta-analysis of hospitalised patients reported that a single episode of delirium was associated with a doubling of mortality rate [3, 4]. Although traditionally considered a transient phenomenon, increasing research shows that delirium can become persistent and is a risk factor for incident dementia [5].

Delirium can be described as prevalent, i.e. found on admission, or incident, when it develops during the hospital admission. Incident delirium is a serious concern in surgical disciplines; delirium rates of over 50% have been reported in older adults undergoing major non-cardiac surgery [4]. Previous research into post-operative delirium (POD) has focused on major orthopaedic or cardiac surgery, with urological patients under-represented [6, 7].

There are reasons to believe that delirium may be a particular issue in Urology. Common urological diseases, including cancers and benign prostatic hyperplasia (BPH), are strongly associated with increasing age which is a generally accepted risk factor for delirium [8, 9]. Urological interventions can be associated with infection, electrolyte disturbance or prescription of anticholinergic drugs—all of which can be triggers to a delirium episode. With changing population demographics and changing expectations of surgery, the urological surgeon is increasingly managing older adults living with frailty and comorbidity, which may further increase the risk of delirium.

A better understanding of urological POD epidemiology and risk factors could inform decisions about treatment. Multicomponent interventions may prevent delirium and if high risk patients could be identified these resources could be applied appropriately [10].

The aim of this study was to identify risk factors for delirium in patients undergoing urological surgery. Systematic reviews of POD in other surgical areas reported small sample sizes and uncertainty in conclusions [6, 11–16]. In this context a comprehensive evidence synthesis can offer the clarity needed to inform practice, research and policy.

## Methods

The preferred reporting items for systematic reviews and meta-analyses (PRISMA) statement guidelines were followed for reporting and the review protocol was prospectively registered with the PROSPERO database (reference CRD42017054613). The study design used in this review was based on previous reviews related to POD in older general and vascular surgical patients [11, 12].

### Search strategy and study eligibility

A comprehensive search strategy was developed using search syntax based on medical subject heading (MeSH) terms and other controlled vocabulary relating to urological surgery, delirium and potential risk factors. The full search strategy is presented in Additional file 1: Fig. 1 (S1) (supporting information). Gastrointestinal and vascular terms were included in the search criteria in case of mixed surgical population studies. It was predicted that such studies would include larger numbers. To ensure these studies were relevant to this review they needed to include at least 50% of patients undergoing urological surgery.

Literature searches were undertaken between January 1987 and June 2019 inclusive. January 1987 was chosen as this coincided with the introduction of the first validated delirium assessment tools [17–19]. Literature searches were conducted across multiple cross-disciplinary electronic databases including: CINAHL® (EBSCO), Embase (Ovid), MEDLINE (Ovid), PSYCinfo® (Ovid) and Web of Science (Thompson Reuters). Citation lists of included studies and relevant reviews were also searched and repeated until no new relevant papers were identified. The grey literature was not assessed. Study selection was performed by two independent authors (AS and IS) and any disagreements were mediated by a third author (JH).

Inclusion criteria were studies of humans published in English, using a validated delirium diagnostic/assessment tool and evaluating risk factors for incident POD. Only full papers published in a peer-reviewed scientific journal were considered. Eligible study designs were primary research evaluating risk factors for incident delirium only, cohort, case–control and cross-sectional studies. The population of interest was patients undergoing elective or emergency urological surgery. The primary outcome of interest was the development of POD (Table 1). The outcome of POD was defined as the proportion of patients experiencing POD following surgery. Exposure(s) for this systematic review were variables or risk factors associated with POD. To develop a provisional set of risk factors to analyze, the NICE delirium guidelines and previous review articles were used [7, 11, 17, 20, 21]. The list was then expanded as additional risk factors were identified. For the full set of risk factors assessed and how they were measured see Additional file 1: Table 1. POD can occur either early or late after surgery, so the duration of follow-up was not defined in the inclusion criteria, but was noted in the analysis, and used to assess risk of bias. Exclusion criteria were studies relating exclusively to delirium tremens and studies based solely in intensive care.

### Quality assessment

An assessment of methodological quality and risk of bias of included studies was conducted by two independent authors using the Newcastle–Ottawa Scale (NOS) [22]. The NOS assesses the design quality of non-randomized studies including case–control and cohort studies. Scores were assigned for selection criteria, comparability and outcome (cohort) or exposure (case–control) with an overall score out of 9. Overall study risk of bias was deemed as high, some concerns or low according to the NOS score (Fig. 2). Studies were deemed to be at high risk of bias overall if any domain (selection criteria, comparability or outcome) received a high risk of bias rating.

### Data analysis

To analyse associations with POD, each risk factor reported in the included studies was recorded with the size of association and statistical significance. Meta-analysis was used to estimate the pooled odds ratio (OR) for dichotomous data, or mean difference (MD) for continuous data, between patients developing POD and those not developing POD. The quality of evidence for each risk factor was assessed using the GRADE criteria and presented in a summary of findings table.

Studies were pooled into a meta-analysis if study designs were considered sufficiently homogeneous and where two or more studies examined the same risk factor in a comparable manner (numerical data available and comparable units of measurement) [23, 24]. Meta-analyses and forest plots were undertaken using Comprehensive meta-analysis (version 3) [25]. A random-effects model was used to pool data. A p value of < 0.05 was considered statistically significant. Statistical heterogeneity was assessed by visual inspection of data and using the Higgins I^2^ statistic, caution was highlighted where I^2^ was greater than 60%.

Sensitivity analyses were conducted based on risk of bias (pre-specified), excluding studies at high risk of bias based on NOS score and overall assessment of bias. Sensitivity analyses were also performed based on heterogeneity of studies in terms of urological operations included.

## Results

### Study selection and incidence

**Fig. 1:**
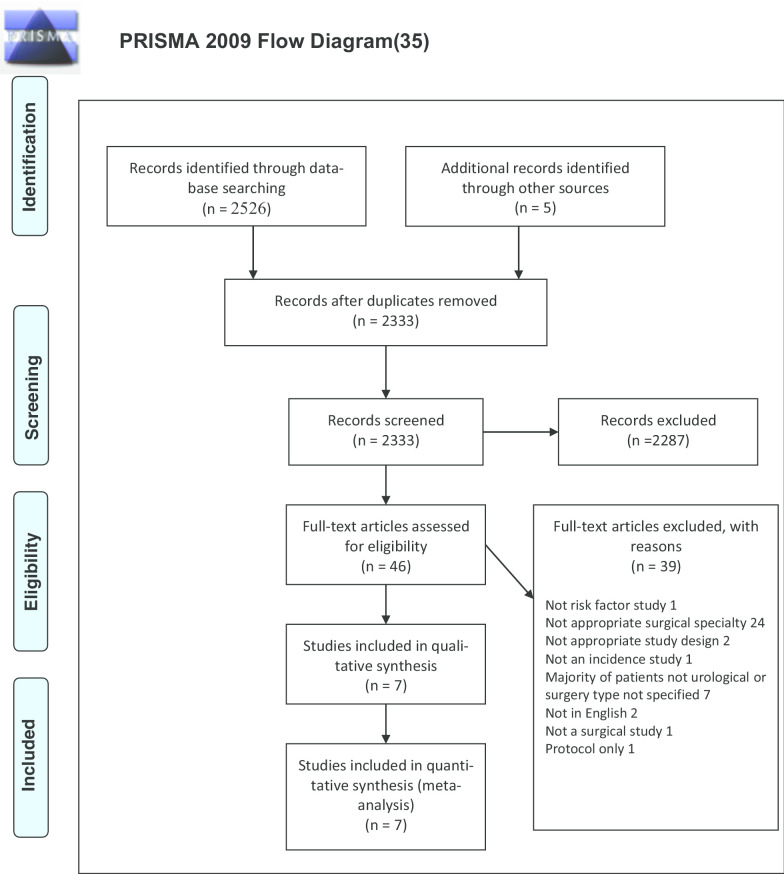
PRISMA flow diagram of results of database literature searching [35]

After removal of duplicates, the initial literature search identified 2331 articles. After title and abstract screening, 44 articles were fully reviewed and 5 met the inclusion criteria [26–30]; see PRISMA flow chart (Fig. 1). A total of 1937 subjects were studied, with 336 (17%) cases of POD. Incidence of POD varied between studies, ranging from 5 to 29%, with the largest study of 640 patients (Gani et al.) having a POD incidence of 26%. Through peer review and further citation searching, we found two new eligible studies [31, 32]. We updated our narrative and quantitative data synthesis to include this new evidence.

### Study characteristics

The main characteristics of the seven included studies are summarised in Table 1. Published between 2005 and 2016, all were prospective cohort studies and studied in patients undergoing urological surgery. Most patients were male (n = 1831, 95%). Studies generally included older adults, one study had an age range 60 years or older [27], four included those 65 years or older [26, 28, 29, 31], another 66 years or older [30]. Finally one study did not restrict inclusion based on age [32]. The overall age for the study population was reported in four studies with a median of 67 years in Sato et al. [32] and the mean age ranging from 71.3 to 74.3 years [27, 29, 30].

**Table 1:** Study characteristics

Study (authors, publication year, country)	Elective/emergency and operation type	Study outcome status: POD/no POD (incidence)	Age: mean (SD)/median (IQR)	Sex: male/female (n)	Criteria for delirium	Assessment frequency (h)	Assessment length (days)	Study overall risk of bias
Large et al. [28]	Elective Radical cystectomy	14/35 (29%)	*Median* With POD 77.8 (73.5–83.5) No POD 73.1 (70.1–76.5)	40/9	CAM	24	3 then days 5, 7	Good
Xue et al. [31]	Elective TURP	28/330 (7.8%)	*Mean* With POD 78.1 (5.33) No POD 74.8 (6.39)	358/0	CAM	24	7	Good
Sato et al. [32]	NS Open and endoscopic surgery	10/205 (4.7%)	*Median* Overall 67 ( 63–75) With POD 79 (77–80) No POD 67 (62–74)	175/40	DSM-V	12–24	3, then daily during hospital LOS	Good
Tognoni et al. [30]	NS Open and TURP > 60mins length	8/82 (8.8%)	*Mean* Overall 74.3 (0.40) With POD 77 (1.7) No POD 74 (0.4)	81/9	CAM, DSM-IV	24	7	Poor
Hamann et al. [27]	Elective Open and endoscopic surgery	7/93 (7.0%)	Overall mean age 71.9 (SD not stated) *Median* With POD 75.1 (IQR not stated) No POD 71.5 (IQR not stated)	77/23	CAM, ICD-10	24	7 or discharge	Poor
Tai et al. [29]	Elective TURP	103/382 (21.2%)	*Mean* 71.3 (2.35)	485/0	CAM, DSM-IV	24	7	Poor
Gani et al. [26]	Elective, all urology patients	166/474 (26.0%)	Median age range 71–75	615/25	CAM	NS	NS	Poor
Totals		336/1601		1831/106		Mean: 24^a^		

*SD *standard deviation, *IQR *interquartile range, *CAM* confusion assessment method, *DSM IV/V *the diagnostic and statistical manual of mental disorders 4th edition/ 5th edition, *ICD 10 *World Health Organization’s International Classification of Diseases version 10, *TURP *transurethral resection of the prostate, *NS *not stated, *LOS *length of stay

^a^Excluding Gani et al.

A range of urological procedures were included, and Table 1 includes a summary of those included in each paper. Three of the included studies were from Europe [26, 27, 30], two based in China [29, 31], one from Japan [32] another from the USA [28]. Six of the included studies used the Confusion Assessment Method (CAM) [33] to screen for delirium, one [32] used DSM-V and one used DSM-IV[30]. Five studies employed CAM once per day for seven days postoperatively [27–30] and one assessed for delirium once or twice daily till discharge from hospital [32]. The seventh study [26], did not specify how frequently or for how long post-operatively they assessed for delirium. Two studies [27, 29] used the World Health Organization’s International Classification of Diseases (ICD-10) criteria [34].

### Study quality

**Fig. 2:**
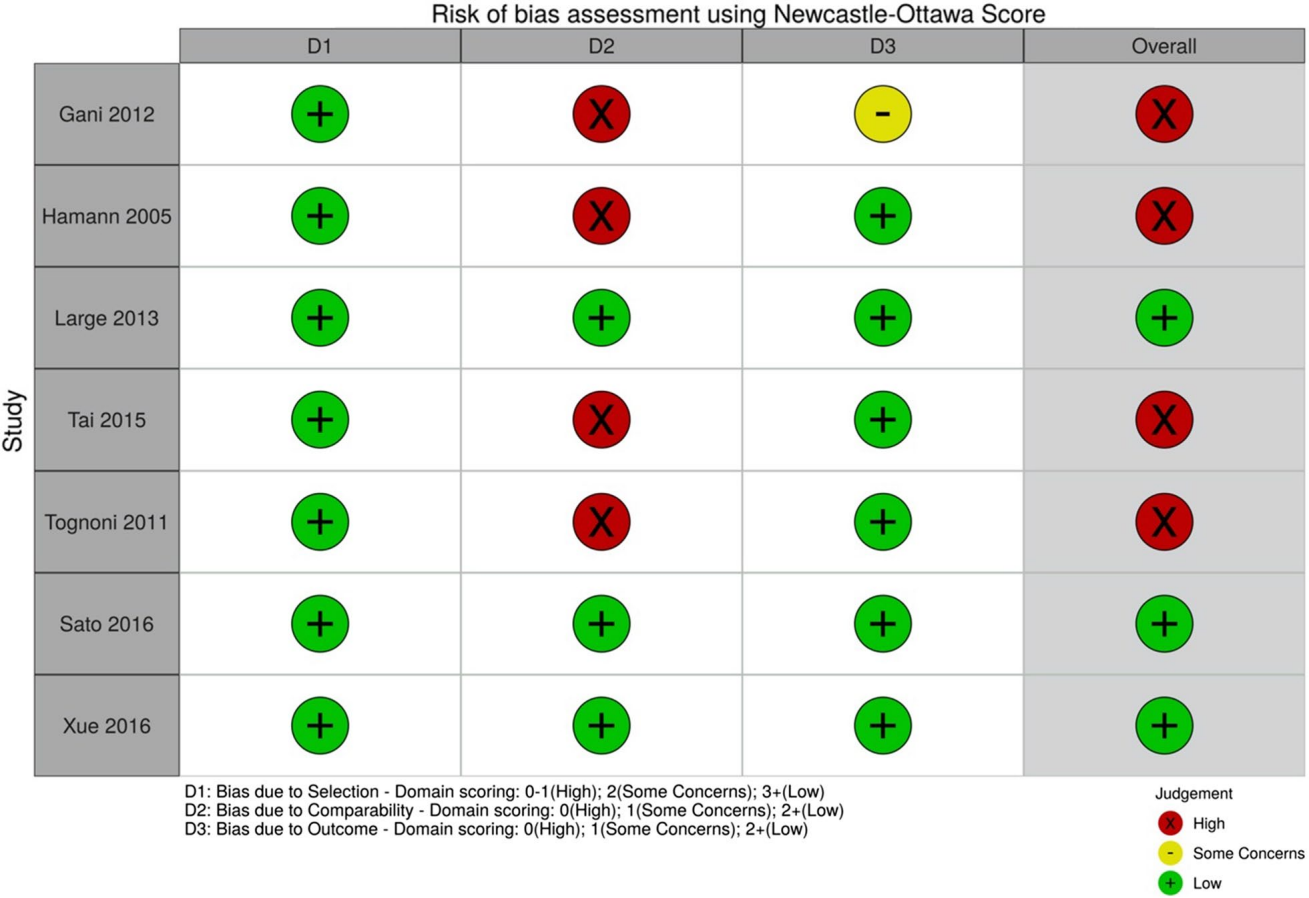
Risk of bias assessment using Newcastle–Ottawa score[22]. Risk of bias assessment for each study according to NOS. Plots created using risk-of-bias visualization (robvis) tool [43]

The seven studies scored between 4 to 9 on the NOS, the majority scoring 7 [27, 29, 30]. Apart from three papers [28, 31, 32], all papers lost two points due to lack of control for age or other factors. Three studies were deemed to be at a low risk of bias overall [28, 31, 32] and four were at high risk of bias overall [26, 27, 29, 30]. The studies at high risk of bias had an overall NOS ranging from 4 to 7 (Fig. 2) [26, 27, 29, 30].

### Risk factors

A total of 26 separate risk factors were studied, with 14 studied in two or more analyses (Table 2). However, only nine risk factors had data suitable for meta-analysis. Six risk factors had pooled results based on data from greater than two studies. The pooled odds ratio (OR, for categorical outcomes) or mean difference (MD, for continuous outcomes) were estimated (Table 3). See Additional file 1: figure (S2) for forest plots.

**Table 2:** Risk factors for post-operative delirium

Risk factor	Hamann 2005 [27]	Large 2012 [28]	Tai 2015 [29]	Tognoni 2010 [30]	Gani 2012 [26]	Sato 2016 [32]	Xue 2016 [31]
*Demographic factors*							
Older age*	=	+	+	+	=	+	+
Male sex ±	=	=		=	=	=	
Married ±		=	=				=
Education length in years				=			=
Mental status (mean MMSE/mean CDT)	=	+	+	= / +			=
History of delirium ±				+			
Depression (DSI > 40 or GDS)	** = **		** + **	** = **			
Comorbidity (based on mean age-adjusted CCI, CCI > 3 or ≥ 2 co-morbidities)	** = **	** = **		** = **			+
Mean BMI		=	=			=	=
ADL (mean score or functions lost)		** = **	** = **	** = **			=
IADL (mean score or functions lost)			+	+			
*Surgical*							
Operation time (mean or median)	+	=				=	
Blood loss (mean or median)		=				=	
Intraoperative hypotension ±				+	?		=
GA versus regional Anaesthesia	=			=		=	=
Alcohol intake (active consumption or excess/abuse)	** = **	** = **	** = **	** = **			=

^*^Age was described by a variety of methods in the included studies such as mean, median or age range. The results presented represent if increasing age was associated with POD

±Proportion of patients with the exposure/risk factor

+ increased risk of POD, = no increased risk of POD, ? not clear due to absence of data. See Additional file 1: Table 1 (S1) for full details

*MMSE* mini mental state examination, *CDT* clock drawing test, *DSI* Depression Status Inventory, *GDS* geriatric depression scale, *CCI* Charlson Comorbidity Index, *BMI *body mass index, *ADL* activities of daily living, *IADL* Instrumental Activities of Daily Living, *GA *general anaesthetic, *CAGE *CAGE questionnaire relates to drinking habits [24]

**Table 3:** Meta-analyses of risk factors for post-operative delirium

Risk factor	Studies/total sample (n/n)	Statistical method	Pooled OR/MD** [95% CI]	Heterogeneity I^2^ (%)
1. Age*	3/663	IV, Random	4.314 [1.597, 7.032]**	73
2. Male Sex	5/1094	M–H, Random	1.284 [0.421, 3.910]	40
3. BMI	4/1107	IV, Random	0.372 [− 0.121, 0.865]**	0
4. ADL score	3/892	IV, Random	0.061 [− 0.776, 0.898]**	98
5. Pre-op MMSE Score	4/982	IV, Random	− 0.476 [− 1.570, 0.618]**	96
6. Education	2/448	IV, Random	− 0.878 [− 1.758, 0.002]**	80
7. CDT mean*	2/575	IV, Random	− 2.443 [− 3.029, − 1.857]**	90
8. Regional anaesthesia	4/763	M–H, Random	0.826, [0.445, 1.533]	0
9. ≥ 2 Co morbidities	2/448	M–H, Random	1.959 [0.984, 3.903]	0

*M−H* Mantel–Haenszel, *IV* inverse variance, *OR * odds ratio, *MD* mean difference, *BMI * body mass index, *ADL* activities of daily living, *MMSE* mini mental state examination, *CDT * clock drawing test

*p-value < 0.05. See Additional file 1: figure 1 for corresponding forest plots.

### Sensitivity and subgroup analyses

Sensitivity analyses were conducted based on study overall risk of bias where possible and studies at a high risk of bias were excluded. This significantly affected the results in the pooled results of two risk factors (Age and ADL) but did not affect the overall result in the other analyses undertaken. Study heterogeneity existed in terms of the operations included within some studies. To assess their impact, sensitivity analyses were performed but the overall result was unchanged in all analyses. A Meta-regression analysis was also considered but due to lack of data and heterogeneity this was not feasible.

### Demographics

#### Age

Age as a risk factor was studied in all the included papers. Five of the studies found older age was a statistically significant risk factor for developing POD [28–32]. One paper found the POD group had an older mean age, but it did not reach statistical significance [27]. The remaining paper examined POD based on age groups, with no significant difference [26]. Pooling of data from three studies [30–32] demonstrated older age was significantly associated with an increased risk of developing POD (Table 3) (MD 4.314; 95% confidence intervals (95% CI) 1.597, 7.032; p = 0.002). A sensitivity analysis based on study risk of bias resulted in a larger mean difference and larger p-value (MD 6.961 95% CI − 1.144, 15.066; p = 0.092). A further sensitivity analysis removing Sato et al. (based on urological operations included in the study) led to narrower CI and a smaller p-value (MD 3.010 95% CI 2.571, 3.448; p < 0.001).

#### Sex

The effect of male sex was examined in all but two papers [29, 31], as these studies only included male participants. Sex was not found to be associated with an increased risk of POD in any study and did not reach statistical significance in the meta-analysis (OR 1.284 95% CI 0.421, 3.910, p = 0.660). The result remained not statistically significant after sensitivity analyses removing studies at high risk of bias (OR 1.147 95% CI 0.358, 3.675; p = 0.817) and removal of three studies [26, 28, 32] based on the urological operations included in these studies (OR 0.649 95% CI 0.103, 4.098; p = 0.645).

#### Marriage

Marriage was recorded in three papers [28, 29, 31], with differing results. One study found an association which was statistically significant [29] whereas the other two found no association.

#### Physical status

Five studies recorded co-morbidities, but only two were comparable as the others used different measurements. The Charlson Co-morbidities Index (CCI) was used in two papers [27, 28] but analysed according to a score ≥ 3 or the mean. Another collectively looked at hyperlipidaemia, hypertension and diabetes as potential risk factors, but no significant difference was found [29]. Two studies used ≥ 2 diseases as a definition of co-morbidity [30, 31]. One of these studies demonstrated a significant association between co morbidities and POD [31], whereas the other did not but did show higher rates of co-morbidity in the POD population [27, 28, 30]. Pooling of results suggests a possible association between having ≥ 2 co-morbidities and an increased risk of POD (OR 1.959 95% CI 0.984, 3.903; p = 0.056).

Meta-analysis of activities of daily living (ADL) scores from three studies found no statistically significant association (MD 0.061 95% CI − 0.776, 0.898; p = 0.886) [28, 29]. Heterogeneity was high in this analysis with an I^2^ of 98%. Sensitivity analysis based on type of surgery did not reach statistical significance (MD 0.227 95% CI − 0.792, 1.246; p = 0.662). Excluding studies at high risk of bias in a further sensitivity analysis resulted in a significantly smaller p-value (MD-0.300 95% CI − 0.514, − 0.086; p = 0.006) and reduced statistical heterogeneity (I^2^ 0%). A poor Instrumental Activities of Daily Living (IADL) score was found to be statistically significant risk factor for POD in two studies [29, 30]. The results were not suitable for meta-analysis as they were not comparable with one study presenting the mean for IADL whereas the other study presented the median score.

The mean BMI of the POD and non-POD groups were comparable in four studies [28, 29, 31, 32] but no association was demonstrated (MD 0.372 95% CI − 0.121 to 0.865; p = 0.139). Sensitivity analyses of pooled data based on operations included in the studies or study risk of bias also did not demonstrate an association.

Only one study [32] assessed risk factors associated with frailty (handgrip strength, get-up and Go test and falls risk assessment score). The authors found an association with all three risk factors.

#### Depression and cognition

Depression was included in three studies, and was found to be associated with POD in one study [29]. Use of anti-depressant medication [27] or psychotropic medication [31] was assessed in two papers but not suitable for pooling and neither found an association with POD. Two of the papers measured depression using the Geriatric Depression Score (GDS) and compared the mean score between the groups [29, 30]. The GDS mean in both POD groups was higher than the non-POD groups, with one study [29] finding an association with a p-value of 0.038 whereas in the other study [30] the p-value was > 0.05 (exact p-value not stated). Results were not pooled due to very high statistical heterogeneity (I^2^ 100%).

Five of the seven studies recorded pre-operative MMSE, but only one found a low score to be associated with an increased risk of POD [28]. Pooled analysis of four study results demonstrated no association (MD − 0.476, 95% CI − 1.570 to 0.618; p = 0.394). Heterogeneity was also high in this analysis with an I^2^ of 96%. Excluding studies at high risk of bias in a sensitivity analysis did not significantly affect the result (MD − 1.104 95% CI − 2.573, 0.365; p = 0.141) but did reduce statistical heterogeneity (I^2^ 67%). A sensitivity analysis based on operations included in the studies also did not demonstrate an association (MD − 0.143 95% CI − 1.315, 1.030; p = 0.811). The majority of the studies excluded patients with a pre-existing history of Alzheimer’s disease. A history of previous delirium was reported statistically significant risk factor for developing POD in one study (Delirium 37.5% vs no Delirium 6%; p = 0.003) [30].

Two studies [29, 30] analysed the association between pre-operative clock drawing test (CDT) and POD. The CDT is an established neuropsychological test of free-hand clock drawing used to screen dementia [36]. Both studies found a significant difference when looking at CDT score as a risk factor for delirium. Tognoni et al. [30] and Tai et al. [29] had similar results; those patients who developed POD had a mean CDT score 2.12 (p = 0.040) and 2.72 (p = 0.038) respectively, less than those who did not develop POD. Pooling of the study results demonstrated a statistically significant association between low CDT score and risk of POD (MD − 2.443 95% CI − 3.029, − 1.857; p < 0.001).

Education, in years, was assessed pre-operatively in two studies [30, 31]. Pooling of the data from the two studies suggests a possible association between shorter education length and an increased risk of POD (MD − 0.878 95% CI − 1.758, 0.002; p = 0.051).

#### Intraoperative factors

No statistically significant impact on risk of developing POD was seen between general and regional anaesthesia. This was the case in individual studies, in the meta-analysis [27, 30–32] (OR 0.826, 95% CI 0.445 to 1.533 p = 0.544) and following sensitivity analyses based on operations included in the study (OR 0.842 95% CI 0.441, 1.607; p = 0.602). Operation time was recorded in three papers, two studies found no association whereas Hamann et al. found longer operation times were associated with POD. [27, 28, 32]. It was not possible to pool the data from these studies. Intra-operative hypotension of less than 90 mmHg systolic, was examined in only one paper and was found to be statistically significant [30]. Xue et al. also assessed hypotension during surgery but it was not possible to pool this data as they did not state how they defined hypotension. They found no association between intra-operative hypotension and POD [31]. Another study mentioned haemodynamic complications appearing to contribute to POD in their 65–70 year old age group, but did not provide data [26].

### Risk of bias across studies

**Fig. 3:**
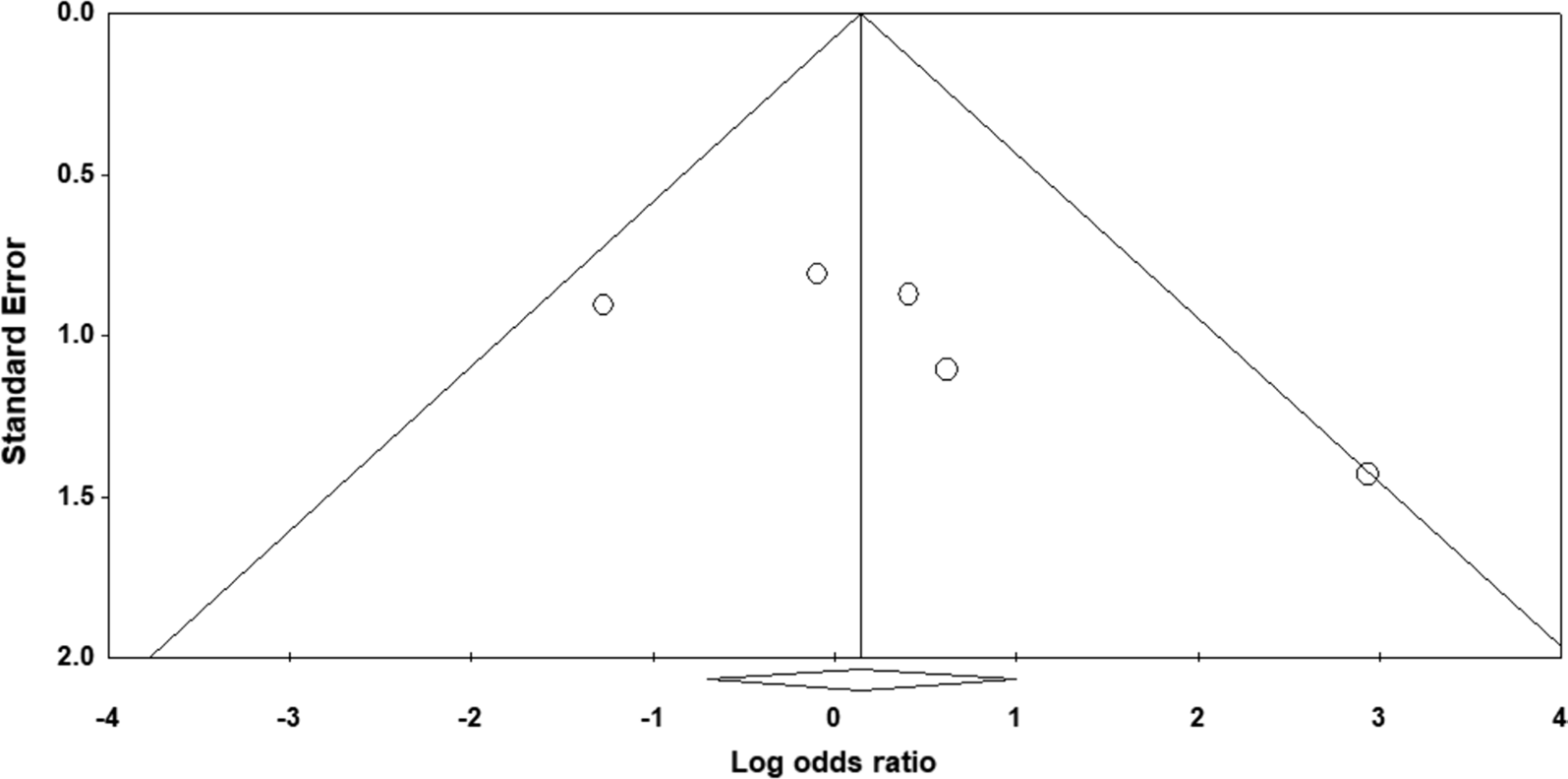
Funnel plot to assess for publication bias. Funnel plot to assess for publication bias[25]

Based on the asymmetry of the funnel plot (Fig. 3) there is a suggestion of publication bias.

### GRADE evaluation of certainty of findings

A 'Summary of findings table' for the nine pooled risk factors analysed within the meta-analysis was created (see Table 4 and Additional file 1: Table 2). The quality of evidence for each outcome was assessed using the five GRADE criteria: risk of bias, consistency of effect, imprecision, indirectness, and other factors including size of effect as well as risk of publication bias (GRADEpro GDT) [37, 38]. Decisions and justifications to down—or upgrade the quality of studies are documented within footnotes.

**Table 4:** GRADE summary of findings table

Certainty assessment							№ of patients		Effect		Certainty	Importance
No. of studies	Study design	Risk of bias	Inconsistency	Indirectness	Imprecision	Other considerations	Delirium	Non-delirium	Relative (95% CI)	Absolute (95% CI)		
*Clock drawing test*												
2	Observational studies	Very serious ^a^	Not serious	Not serious	Not serious	None	111	464	−	MD 2.443 lower (3.029 lower to 1.857 lower)	⨁◯◯◯ VERY LOW	IMPORTANT
*Age*												
3	Observational studies	Very serious ^c^	Not serious	Not serious	Serious ^b^	None	46	617	−	MD 4.314 higher (1.597 higher to 7.032 higher)	⨁◯◯◯ VERY LOW	IMPORTANT

Bibliography: Tai et al. [29], Tognoni et al. [30], Sato et al. [32], Xue et al. [31]

*CI* confidence interval, *MD* mean difference, *OR* odds ratio

*p-value < 0.05. See Additional file 1: Fig. 1 for corresponding forest plots

*Explanations*

a. Downgraded as both studies did not control for age or other factors associated with delirium

b. Wide confidence intervals

c. Downgraded as all 3 studies did not control for age or other factors associated with delirium

Based on the GRADE certainty of evidence assessment, all nine risk factors had a very low certainty of evidence.

## Discussion

Seven studies [26–30] were identified for inclusion in this systematic review assessing risk factors associated with POD in urological surgical patients. The included studies had a predominantly elderly male patient population and analysed a variety of risk factors for their association with delirium post operatively. The 16 broad risk factors were reported using 26 different methods and studies included patients undergoing very different operations, allowing only nine risk factors to undergo meta-analysis; the majority only containing data from 2 studies. Of the risk factors included in meta-analysis, the clock drawing test and age were the only two that reached statistical significance.

Although the other risk factors were not found to be significantly associated with delirium, it is important to interpret these with caution as four of the included studies were deemed to be at a high risk of bias. In addition, the majority of risk factors assessed within the meta-analysis contained data from two studies. Meta-analysis was limited by the heterogeneity of the data and the types of operations included, with a number of risk factors unable to be pooled or limited studies for the meta-analysis. These included co-morbidities and IADLs, both found within the individual studies to be associated with the development of post-operative delirium.

With regard to co-morbidity, this was higher within the delirium group in four of the studies [27, 28, 30, 31] with one study finding a significant association [31]. Pooling was only possible for two studies (two or more co-morbidities). The result suggests that having two or co-morbidities is associated with POD, although it did not achieve our cut off for statistical significance (p = 0.056) and included one study at high risk of bias. This could be either to true lack of association or due to lack of statistical power. Therefore, research with a larger cohort of patients, using the same assessment method and possibly looking at specific co-morbidities such as dementia, depression and visual/hearing impairment would be of use. A shorter duration of education also appears to be associated with an increased risk of POD despite again not achieving significance within the meta-analysis (p = 0.051) possibly for the same reasons as co-morbidity.

The two significant results in the meta-analysis were a lower mean CDT score and higher mean age in those who developed POD. These results should be interpreted with caution as both analyses included studies at high risk of bias. The sensitivity analysis conducted, based on study risk of bias, also suggests the result for the association between older age and POD is not very robust and limits its interpretation. Despite this, advancing age is a well-recognised risk factor for delirium and similar associations have been demonstrated within the literature [12–15, 39]. CDT is a screening tool for cognitive impairment and dementia [36], and the association with delirium may justify its use to establish underlying cognitive impairment preoperatively and delirium risk postoperatively [20]. Finally, ADL was not initially found to be associated with POD, but after excluding studies at high risk of bias, there appeared to be an association. However, its interpretation is limited due to small study sample sizes.

Numerous systematic reviews have been undertaken in both surgical and non-surgical patient populations. Reviews in post-operative surgical patients have mainly focused on Vascular [12], Gastrointestinal [11], Cardiac [13, 14] and Orthopaedic [6, 15, 16] specialties, with incidence ranges (4%-55%) aligning with the results from this systematic review. Incidence ranges are also similar in the medical inpatient setting [17, 40], but significantly higher within intensive care [39, 41]. A multitude of risk factors have been analysed via meta-analyses or multivariable analyses for an association with incident delirium. Most commonly, increasing age [12–15, 39], cognitive impairment [6, 13, 14, 16] and alcohol excess [11, 39] have been identified to increase the risk of developing delirium. Other factors were more mixed, similar to results observed within this review, such as BMI and sex [12, 15–17, 39].

These previous systematic reviews on incident delirium have also highlighted the heterogeneity in the risk factors analysed within the included studies and difficulties pooling results in a similar manner to this review. As discussed above, a low mean CDT score was identified within this review to be associated with POD, a result not replicated within the current body of systematic reviews. Although not technically a risk factor, it does present a potential screening tool to identify those at risk of delirium who could then be targeted for interventions to reduce the risk of POD occurring. Although promising, these results do have limitations and therefore the CDT would need extensive further evaluation before use as a screening tool within clinical practice. A recently published prospective study of over 1000 patients identified a different cognitive screening tool for dementia was associated with development of POD [42]. The authors found that a Hasegawa Dementia Scale-Revised (HDS-R) score of less than 20 was an independent risk factors for POD in elderly urological patients [42]. However, they also identified that its use for all urological patients would be limited as only 3% of patients with this as their only risk factor developed POD [42]. These considerations would need to be taken into account for CDT also.

This review does have a number of limitations. The main one being that there are relatively few studies on this subject within the literature and the numbers of patients within those studies are relatively small. Therefore, the lack of association found may be a result of a lack of statistical power rather than there being no true association. The largest study was not well detailed [26] and the majority of studies are at a high risk of bias. The heterogeneity of the risk factors studied and inconsistency between the studies in how the data were recorded makes it difficult to fully assess the various risk factors. In terms of limitations of the studies themselves, the major issue was with assessment of delirium. This occurred once per day in the majority of the studies which, in view of the fact delirium has a fluctuating course, may mean some cases were missed and thus the true incidence of post-operative delirium is likely to have been under reported. A final limitation is that two of the studies excluded patients with Alzheimer's and dementia without adequate explanation. This is especially important, as dementia is known to be a risk factor for the development of delirium [14].

This review has highlighted that there is a lack of research in post-operative delirium in urological patients and within the relevant studies there is heterogeneity between the risk factors assessed, often with small numbers of patients. Importantly, this review has identified a number of potential areas for future research. A number of statistically significant risk factors in individual studies, including MMSE, CDT, depression, IADL functions, previous delirium, severity of urological disease, duration of surgery and intra-operative hypotension, were reported as being associated with the development of post-operative delirium. To improve our understanding of delirium in this group of patients, future studies should focus on comparable risk factors and methods of data collection as well as possible collaborative work.

## Conclusion

In summary, research into post-operative delirium within the urological surgical population is limited. Of those studies included in this systematic review, there is high risk of bias and the heterogeneity of risk factors assessed was restrictive to pooling of data and meta-analysis. It has, however, raised a number of risk factors worthy of further research, and highlighted the importance of future collaborative and comparative work to increase our understanding of risk factors associated with post-operative delirium within the urological patient population.

## Supplementary information

**Additional file 1**. Supplementary information containing the search strategy (S1), meta-analysis forest plots (S2), extended risk factor table (supplementary Table 1) and extending GRADE summary of findings table (supplementary Table 2).

## Data Availability

All data generated or analysed during this study are included in this published article [and its supplementary information files].

## Abbreviations

POD: Post-operative delirium
NOS: Newcastle–Ottawa scale
OR: Odds ratio
MD: Mean difference
CI: Confidence interval
CDT: Clock drawing test
DSM: The diagnostic and statistical manual of mental disorders
BPH: Benign prostatic hyperplasia
PRISMA: Preferred reporting items for systematic reviews and meta-analyses
NICE: National Institute for Health and Care Excellence
MeSH: Medial subject heading
CAM: Confusion assessment method
ICD: World Health Organisation’s International Classification of Diseases
MMSE: Mini mental state examination
CCI: Charlson Co-morbidities Index
ADL: Activities of daily living
IADL: Instrumental Activities of Daily Living
BMI: Body mass index
GDS: Geriatric Depression Score
DSI: Depression Status Inventory
GA: General anaesthetic
SD: Standard deviation
IQR: Interquartile range
TURP: Trans-urethral resection of the prostate
M-H: Mantel Haenszel
IV: Inverse variance
