# Supplementary material for: Risk factors for incident delirium among urological patients: a systematic review and meta-analysis with GRADE summary of findings
Source: BMC Urol. 2020 Oct 27;20:169. doi: 10.1186/s12894-020-00743-x (PMC7590461; doi:10.1186/s12894-020-00743-x)
Supplement: Supplementary file 1 — Additional file 1. Supplementary information containing the search strategy (S1), meta-analysis forest plots (S2), extended risk factor table (supplementary Table 1) and extending GRADE summary of findings table (supplementary Table 2). [file 12894_2020_743_MOESM1_ESM.docx]

# Supplementary material

1. Delirium/

2. Delirium, Dementia, Amnestic, Cognitive Disorders/

3. "acute confus*".mp.

4. "acute organic psychosyndrome".mp.

5. "acute organic psychosyndrome*".mp.

6. "metabolic encephalopath*".mp.

7. "acute psycho-organic syndrome*".mp.

8. "clouded state*".mp.

9. "clouding of consciousness".mp.

10. "exogenous psycho*".mp.

11. "toxic psycho*".mp.

12. "toxic confusion*".mp.

13. 1 or 2 or 3 or 4 or 5 or 6 or 7 or 8 or 9 or 10 or 11

14. Risk Factors/

15. Risk Assessment/

16. "predictive value of tests".mp.

17. prognos*.mp.

18. sensitivity.mp.

19. specificity.mp.

20. "ROC curve*".mp.

21. "predictive value*".mp.

22. prediction.mp.

23. "decision support technique*".mp.

24. "exp* decision*".mp.

25. "decision aid*".mp.

26. "decision analysis".mp.

27. "decision model*".mp.

28. "decision support".mp.

29. causality/

30. 14 or 15 or 16 or 17 or 18 or 19 or 20 or 21 or 22 or 23 or 24 or 25 or 26 or 27 or 28 or 29

31. exp Specialties, Surgical/

32. operation*.mp.

33. post-op*.mp.

34. postop*.mp.

35. preop*.mp.

36. pre-op*.mp.

37. periop*.mp.

38. peri-op*.mp.

39. anaes*.mp.

40. 31 or 32 or 33 or 34 or 35 or 36 or 37 or 38 or 39

41. 13 and 30 and 40

42. urol*.mp.

43. gen*.mp.

44. vasc*.mp.

45. colo*.mp.

46. (gastrointestinal or gastro-intestinal).mp.

47. 42 or 43 or 44 or 45 or 46

48. 13 and 30 and 41 and 47

49. limit 53 to yr="1987 -Current"

**Supplementary figure 1 (S1) Search strategy for MEDLINE.**

Search strategy was initially conducted in MEDLINE (Ovid), then adapted to the other databases.

**Age**

**
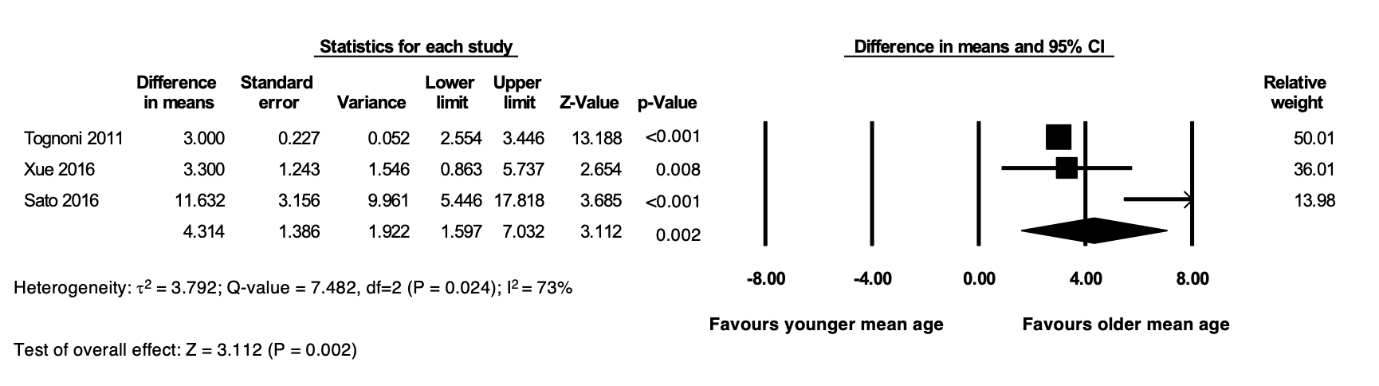
**

**BMI**

τ

2

τ

2

τ

2

τ

2


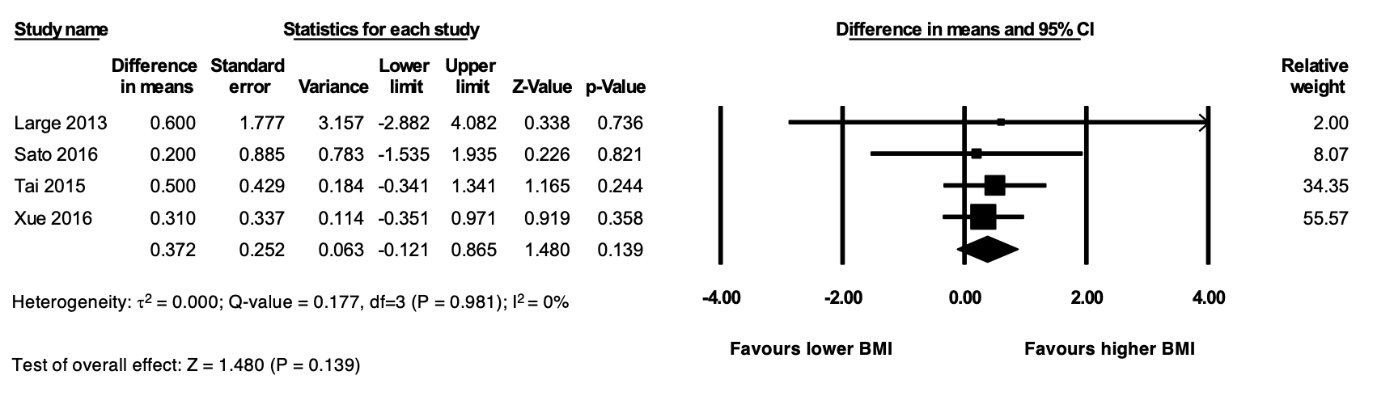


**Mean ADL Score**


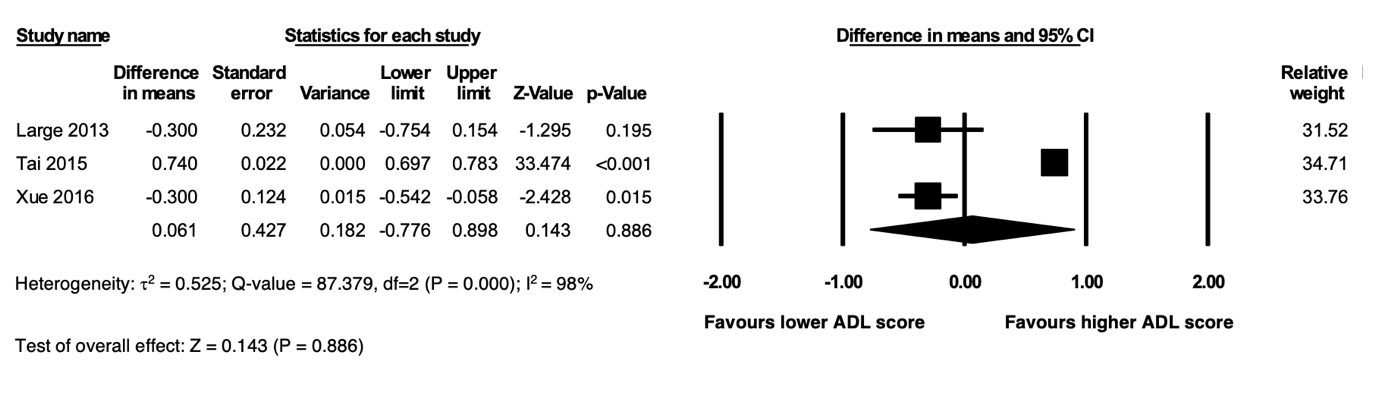


**Mean MMSE**


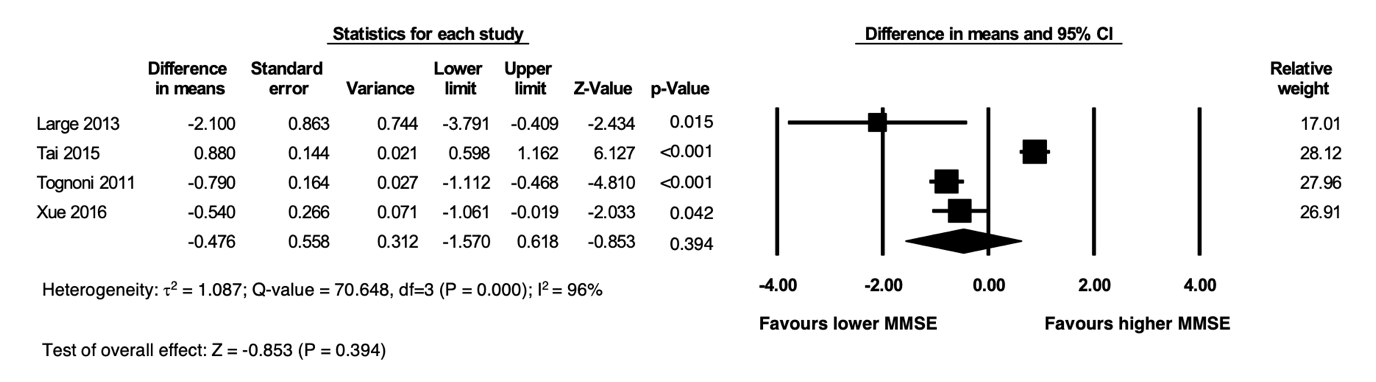


**Education length (years)**


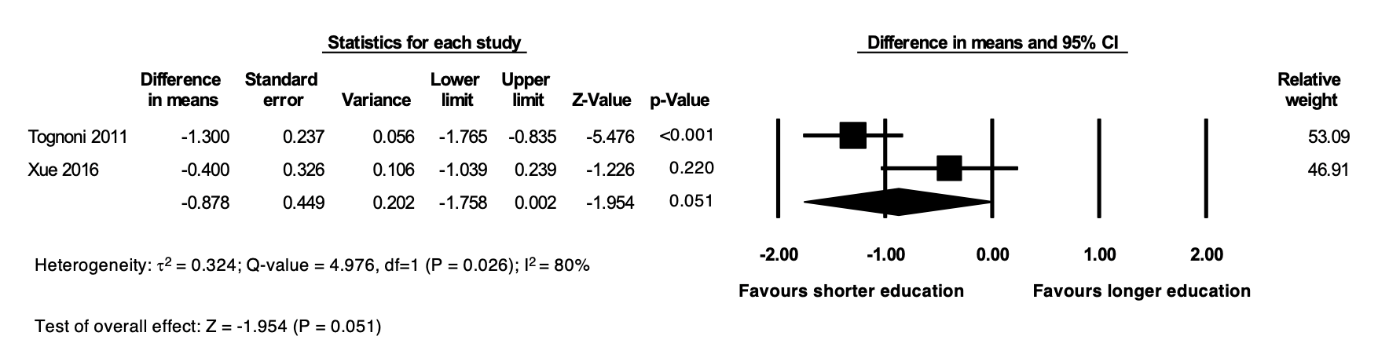


**Clock drawing test (CDT)**


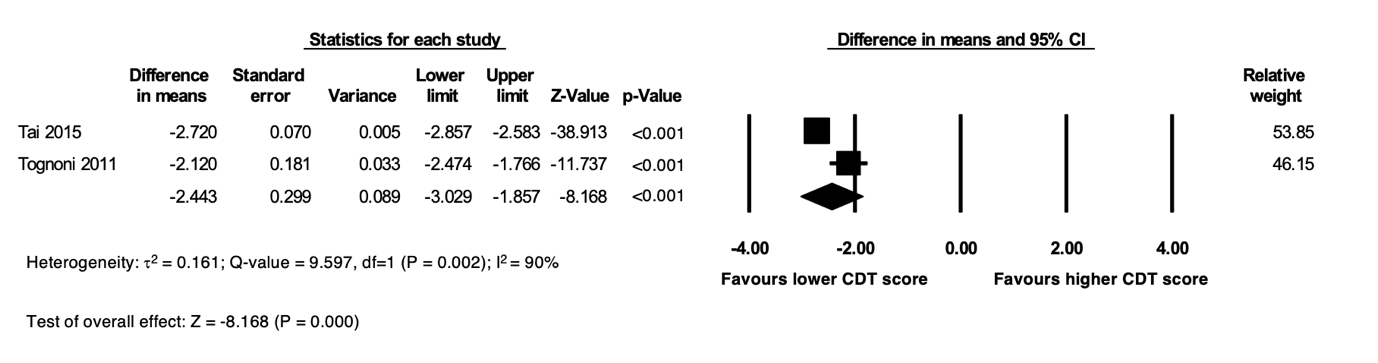


**Male sex**


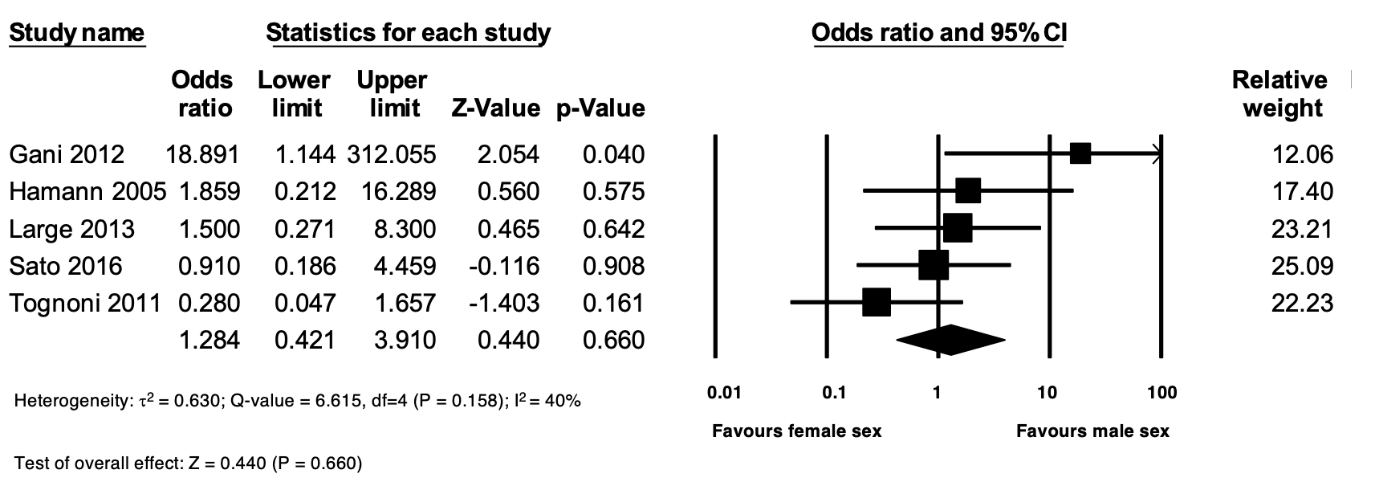


**≥ 2 co-morbidities**


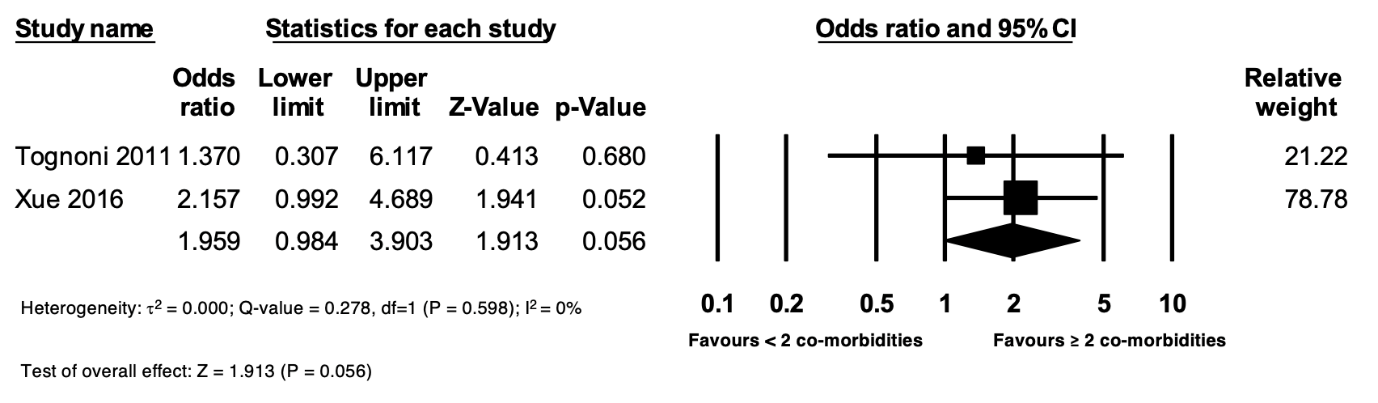


**Regional Anaesthesia**


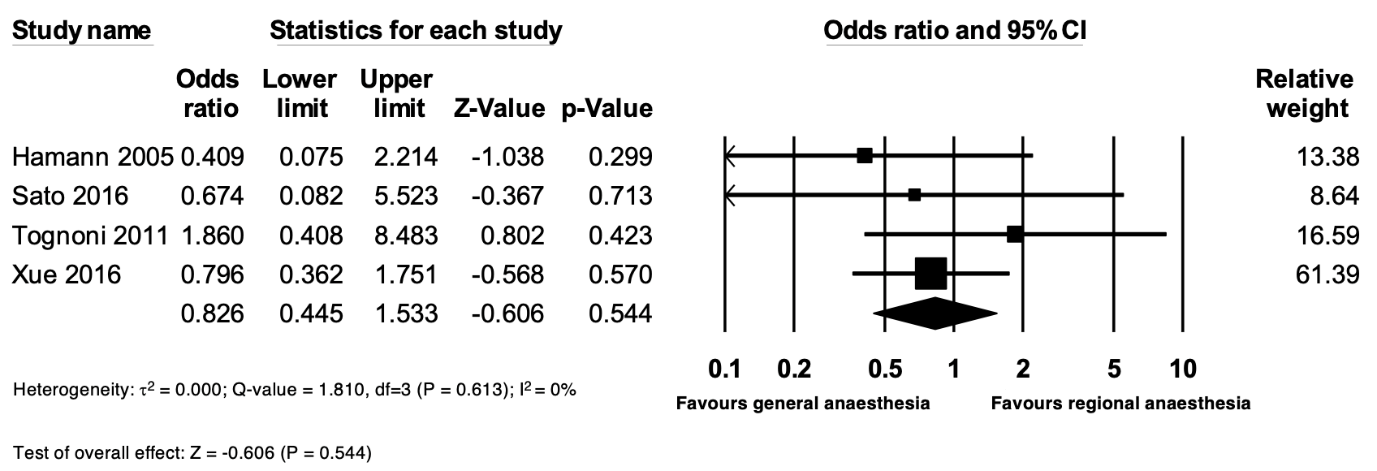


**Supplementary figure 2 (S2). Meta-analysis forest plots**

Forest plots for the nine risk factors included within the meta-analysis.

| **Risk Factor** | **Hamann 2005 (**[**27**](#_ENREF_27)**)** | **Large**  **2012 (**[**28**](#_ENREF_28)**)** | **Tai**  **2015 (**[**29**](#_ENREF_29)**)** | **Tognoni**  **2010 (**[**30**](#_ENREF_30)**)** | **Gani**  **2012 (**[**26**](#_ENREF_26)**)** | **Sato 2016 (**[**32**](#_ENREF_32)**)** | **Xue 2016 (**[**31**](#_ENREF_31)**)** |
| --- | --- | --- | --- | --- | --- | --- | --- |
| **Demographic Factors** | | | | | | | |
| Older Age * | = | + | + | + | = | + | + |
| Male sex ± | = | = |  | = | = | = |  |
| Married ± |  | = | - |  |  |  | = |
| Education length in years |  |  |  | = |  |  | = |
| **Mental Status** | | | | | | | |
| Mean MMSE | = | + | + | = |  |  | = |
| Mean CDT |  |  | + | + |  |  |  |
| History of delirium ± |  |  |  | + |  |  |  |
| **Depression (Combining 2 risk factors below)** | **=** |  | **+** | **=** |  |  |  |
| Depression (DSI>40) ± | = |  |  |  |  |  |  |
| Mean GDS |  |  | + | = |  |  |  |
| **Physical Illness** | | | | | | | |
| **Comorbidity (Combining 3 risk factors below)** | **=** | **=** |  | **=** |  |  | **+** |
| Mean CCI |  | = |  |  |  |  |  |
| CCI >3 ± | = |  |  |  |  |  |  |
| 2 or more Co-morbidities ± |  |  |  | = |  |  | + |
| Mean BMI |  | = | = |  |  | = | = |
| **Physical Status** | | | | | | | |
| Mean ADL |  | = | = |  |  |  | = |
| ADL functions lost |  |  |  | = |  |  |  |
| Mean IADL |  |  | + |  |  |  |  |
| IADL functions lost |  |  |  | + |  |  |  |
| **Surgical** | | | | | | | |
| Operation time | + | = |  |  |  | = |  |
| Blood loss |  | = |  |  |  | = |  |
| Intraoperative hypotension ± |  |  |  | + | ? |  | = |
| Regional Anaesthesia (versus General Anaesthesia) | = |  |  | = |  | = | = |
| **Alcohol (combining 4 risk factors below)** | **=** | **=** | **=** | **=** |  |  | = |
| CAGE >1 ± | = |  |  |  |  |  |  |
| >14 Drinks per week ± |  | = |  |  |  |  |  |
| Average alcohol consumption |  |  | = |  |  |  | = |
| Alcohol Abuse ± |  |  |  | = |  |  |  |

* Age was described by a variety of methods in the included studies such as mean, median or age range. The results presented represent if increasing age was associated with POD.

± proportion of patients with the exposure/risk factor.

**Supplementary table 1. Extended table of risk factors for Post-operative delirium.**

**Key:** MMSE - mini mental state examination, CDT – clock drawing test, DSI - Depression Status Inventory, GDS - geriatric depression scale, CCI - Charlson Comorbidity Index, BMI – Body mass index, ADL - activities of daily living, IADL - Instrumental Activities of Daily Living, CAGE – CAGE questionnaire relates to drinking habits (24), + increased risk of POD, = no increased risk of POD, ? not clear due to absence of data.

**Question**: Pre-operative risk factors associated with incident delirium

**Setting**: Inpatient

**Bibliography**: Large et al. ([28](#_ENREF_28)), Tognoni et al. ([30](#_ENREF_30)), Hamann et al. ([27](#_ENREF_27)), Tai et al. ([29](#_ENREF_29)), Gani et al. ([26](#_ENREF_26)) Sato et al. ([32](#_ENREF_32)) and Xue et al. ([31](#_ENREF_31))

| **Certainty assessment** | | | | | | | **№ of patients** | | **Effect** | | **Certainty** | **Importance** |
| --- | --- | --- | --- | --- | --- | --- | --- | --- | --- | --- | --- | --- |
| **№ of studies** | **Study design** | **Risk of bias** | **Inconsistency** | **Indirectness** | **Imprecision** | **Other considerations** | **Delirium** | **Non-delirium** | **Relative (95% CI)** | **Absolute (95% CI)** |  |  |
| **Clock Drawing Test** | | | | | | | | | | | | |
| 2 | observational studies | very serious ^a^ | not serious | not serious | not serious | none | 111 | 464 | - | MD **2.443 lower** (3.029 lower to 1.857 lower) | ⨁◯◯◯ VERY LOW | IMPORTANT |
| **Male sex** | | | | | | | | | | | | |
| 5 | observational studies | very serious ^b^ | not serious | not serious | serious ^c^ | none | 198/205 (96.6%) | 800/889 (90.0%) | **OR 1.284** (0.421 to 3.910) | **20 more per 1,000** (from 109 fewer to 72 more) | ⨁◯◯◯ VERY LOW | IMPORTANT |
| **Body Mass Index (BMI)** | | | | | | | | | | | | |
| 4 | observational studies | very serious ^d^ | not serious | not serious | serious ^c^ | none | 155 | 952 | - | MD **0.372 higher** (0.121 lower to 0.865 higher) | ⨁◯◯◯ VERY LOW | IMPORTANT |
| **Mean Activities of daily living (ADL) score** | | | | | | | | | | | | |
| 3 | observational studies | very serious ^d^ | not serious | not serious | serious ^c^ | none | 145 | 747 | - | MD **0.061 higher** (0.776 lower to 0.898 higher) | ⨁◯◯◯ VERY LOW | IMPORTANT |
| **Pre-op Mean MMSE score** | | | | | | | | | | | | |
| 4 | observational studies | very serious ^e^ | not serious | not serious | serious ^c^ | none | 153 | 829 | - | MD **0.476 lower** (1.57 lower to 0.618 higher) | ⨁◯◯◯ VERY LOW | IMPORTANT |
| **Regional Anaesthesia** | | | | | | | | | | | | |
| 4 | observational studies | very serious ^a^ | not serious | not serious | serious ^c^ | none | 17/53 (32.1%) | 243/710 (34.2%) | **OR 0.826** (0.445 to 1.533) | **42 fewer per 1,000** (from 228 fewer to 240 more) | ⨁◯◯◯ VERY LOW | IMPORTANT |
| **Education (years)** | | | | | | | | | | | | |
| 2 | observational studies | very serious ^a^ | not serious | not serious | serious ^c^ | none | 36 | 412 | - | MD **0.878 lower** (1.758 lower to 0.002 higher) | ⨁◯◯◯ VERY LOW | IMPORTANT |
| **Age** | | | | | | | | | | | | |
| 3 | observational studies | very serious ^e^ | not serious | not serious | serious ^c^ | none | 46 | 617 | - | MD **4.314 higher** (1.597 higher to 7.032 higher) | ⨁◯◯◯ VERY LOW | IMPORTANT |
| **≥ 2 co-morbidities** | | | | | | | | | | | | |
| 2 | observational studies | very serious ^a^ | not serious | not serious | serious ^c^ | none | 20/36 (55.6%) | 160/412 (38.8%) | **OR 1.959** (0.984 to 3.903) | **166 more per 1,000** (from 4 fewer to 324 more) | ⨁◯◯◯ VERY LOW | IMPORTANT |

**Supplementary table 2 (S2) GRADE Summary of findings table for nine pre-operative delirium risk factors.**

**CI:** Confidence interval; **MD:** Mean difference; **OR:** Odds ratio

#### Explanations

a. downgraded as both studies did not control for age or other factors associated with delirium

b. downgraded as 3 studies did not control for age or other factors associated with delirium. Also Gani et al. at high risk of bias as limited detail on follow up and ensuring delirium not present at start of study.

c. wide confidence intervals

d. downgraded as Tai et al. and Xue et al. did not control for age or other factors associated with delirium

e. downgraded as 3 of the studies did not control for age or other factors associated with delirium
